# Robustly photogenerating H_2_ in water using FeP/CdS catalyst under solar irradiation

**DOI:** 10.1038/srep19846

**Published:** 2016-01-28

**Authors:** Huanqing Cheng, Xiao-Jun Lv, Shuang Cao, Zong-Yan Zhao, Yong Chen, Wen-Fu Fu

**Affiliations:** 1 Key Laboratory of Photochemical Conversion and Optoelectronic Materials and HKU-CAS Joint Laboratory on New Materials, Technical Institute of Physics and Chemistry, Chinese Academy of Sciences, Beijing 100190, P. R. China; 2 College of Chemistry and Chemical Engineering, Yunnan Normal University, Kunming 650092, P. R. China; 3 Faculty of Materials Science and Engineering, Kunming University of Science and Technology, Kunming 650093, P. R. China

## Abstract

Photosplitting water for H_2_ production is a promising, sustainable approach for solar-to-chemical energy conversion. However, developing low-cost, high efficient and stable photocatalysts remains the major challenge. Here we report a composite photocatalyst consisting of FeP nanoparticles and CdS nanocrystals (FeP/CdS) for photogenerating H_2_ in aqueous lactic acid solution under visible light irradiation. Experimental results demonstrate that the photocatalyst is highly active with a H_2_-evolution rate of 202000 μmol h^−1^ g^−1^ for the first 5 h (106000 μmol h^−1^ g^−1^ under natural solar irradiation), which is the best H_2_ evolution activity, even 3-fold higher than the control *in situ* photo-deposited Pt/CdS system, and the corresponding to an apparent quantum efficiency of over 35% at 520 nm. More important, we found that the system exhibited excellent stability and remained effective after more than 100 h in optimal conditions under visible light irradiation. A wide-ranging analysis verified that FeP effectively separates the photoexcited charge from CdS and showed that the dual active sites in FeP enhance the activity of FeP/CdS photocatalysts.

The production of chemical fuels using sunlight is an attractive and sustainable solution to global energy and environmental problems^1^,^2^. Since the 1970s, splitting water using solar energy has received much attention as a possible means for converting solar energy to chemical energy by creating clean and renewable hydrogen fuel^3^,^4^. Molecular hydrogen (H_2_) production using semiconductor photocatalysts is one of the most promising strategies for light-driven proton reduction^5^,^6^,^7^. However, most semiconductors cannot produce H_2_ without a co-catalyst, even in the presence of sacrificial electron donor. This is attributed to the quick recombination of electron and hole pairs while migrating to the surface, and the surface reaction being too slow to efficiently consume these charges^3^. Generally, to prevent the recombination of electron and hole pairs, co-catalysts (such as metals and especially noble metals) are used to serve as electron sinks and provide effective proton-reduction reaction sites^8^. Platinum (Pt) is the most widely used co-catalyst for the photocatalytic production of H_2_ from water because of its high activity and stability under the often harsh operational conditions. However, noble metals like Pt are expensive and scarce. It is therefore useful to develop high efficiency, low-cost, noble-metal-free co-catalysts to further facilitate the development of H_2_ photogeneration. Several new earth-abundant metal compounds have emerged and can be good candidates for co-catalysts, including MoS_2_^9^,^10^,^11^,^12^, NiS^13^,^14^, NiS_x_^15^, CuS^16^, Cu(OH)_2_^17^, Co(OH)_2_^18^, and other related materials^19^. However, these co-catalysts also have the drawback of instability during the photocatalytic reaction. Very recently, metal phosphides, such as Ni_2_P^20^, CoP^21^,^22^, CuP^23^, MoP^24^, and FeP^25^,^26^,^27^,^28^ have been found to have the high electrochemical catalysis activity and good stability for the hydrogen evolution reaction (HER) in acid or alkali solutions. Transition metal phosphides, which involve the alloying of metals and phosphorus (P), have demonstrated high activity for the HER and hydrodesulfurization reactions because of their ability to reversibly bind hydrogen. However, the photocatalytic activity of these metal phosphides as the co-catalyst for H_2_ production has not yet been fully explored. We recently reported that a colloidal metal phosphide (Ni_2_P or Co_2_P) catalyst combined with colloidal CdS nanorod photosensitizers displayed good photocatalytic H_2_ evolution activity in an aqueous lactic acid solution, revealing the co-catalyst potential of metal phosphides^29^,^30^. Iron-based alternatives are especially attractive because Fe is the most abundant transition metal and its price is typically at least two orders of magnitude less than that of other highly abundant and catalytically relevant metals, including Ni and Co^25^. Iron phosphide (FeP) nanoparticles (NPs) as co-catalysts deposited on TiO_2_ have been shown to be exceptionally active for sustained H_2_ production in either acidic or neutral-pH aqueous solutions under UV light irradiation^25^. However, highly active photocatalysts composed of high-quality, iron-based nanoparticulate materials under visible light irradiation are among the most desired because of their low cost, abundance, and ease of processing.

Herein we performed a noble-metal-free system of visible-light driven H_2_ production with the best activity and significant longevity. The system includes the semiconductor CdS and a FeP composite photocatalyst that together exhibit high activity and good photochemical stability under artificial and natural irradiation. The essence of the thermodynamic relationship between CdS and FeP is elucidated, and the mechanism of effective charge separation based on the band alignment in such system is also studied in depth. This information will be useful for providing insight for the design and preparation of efficient semiconductor-based photocatalysts.

## Results

### Characterization of FeP samples

FeP nanoparticles were prepared by chemical conversion from Fe_3_O_4_ nanoparticles precursor (Figs S1–S3) via the low-temperature phosphidation reaction under Ar atmosphere. FeP adopted a hexagonal structure (Fig. S4a) and is a well-known electrochemical hydrogen evolution catalyst^25^,^28^,^31^. The structure of the FeP (103) surface results in Fe and P sites being simultaneously exposed (Fig. S4b). This resulted in an ensemble effect, whereby proton-acceptor and hydride-acceptor centres are both present to facilitate catalysis of the HER^20^.

**Figure 1:**
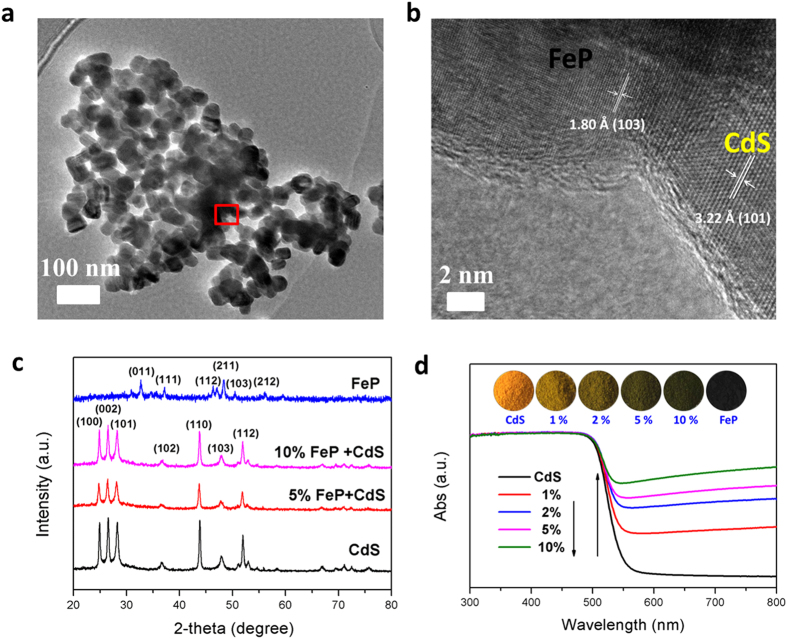
Characterization of FeP/CdS samples. (**a**) TEM image of 5wt% FeP/CdS composite photocatalysts. (**b**) HRTEM image taken from the area marked with a red rectangle in (**a**). (**c**) XRD patterns for samples of pure CdS and for those with various amounts of FeP co-catalysts. (**d**) UV-vis spectra of pure CdS samples and for those with various amounts of FeP co-catalysts.

The intense grinding of the various ratios of FeP and CdS ensured the formation of a robust solid-solid interface between the FeP NPs and the CdS supports. Figure 1a shows transmission electron microscope (TEM) images of 5 wt% FeP/CdS photocatalysts. A high-resolution TEM (HRTEM) image clearly reveals the interaction between the NPs of FeP and CdS (Fig. 1b). The well-resolved lattice fringes with distances of 1.8 and 3.22 Å correspond to the (103) and (101) planes for FeP and CdS, respectively. X-ray diffraction (XRD) results showed no clear FeP peaks after loading 5 wt% and 10 wt% FeP on the CdS (Fig. 1c). This lack of peaks may be attributed to effective dispersion and a lack of crystallization of FeP, which together may have led to a relatively low diffraction intensity of the FeP on the CdS^32^,^33^,^34^. Figure 1d shows the UV-vis diffuse reflectance spectra of samples of pure CdS and those when loaded with different amounts of FeP co-catalyst. Pure CdS absorbed visible light with wavelengths around 557 nm and a corresponding band gap of 2.2 eV. CdS samples gradually increased their visible light absorption as the amount of FeP co-catalysts increased. This is attributed to FeP’s deep black colour being beneficial to photocatalytic activity.

### Photocatalytic activity

**Figure 2:**
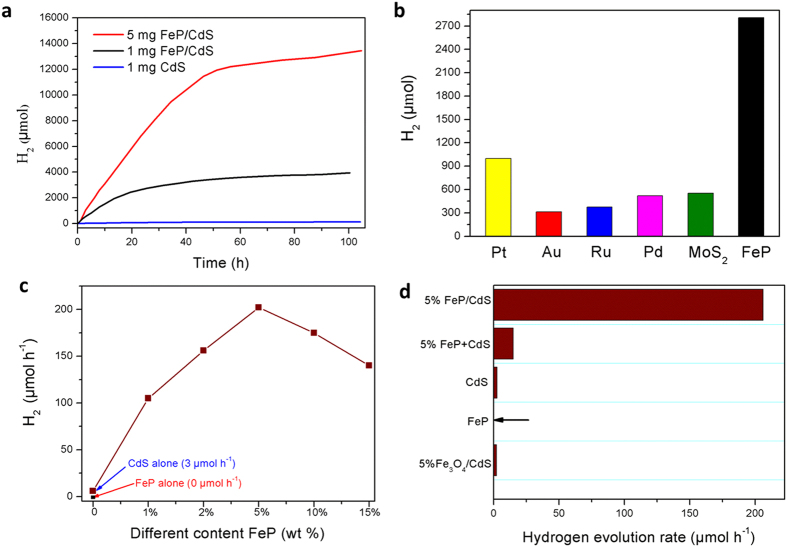
Photocatalytical activity comparison. (**a**) Photocatalytic activity of H_2_ evolution over time from 5 wt% FeP under visible light irradiation at pH 2.0 solution. (**b**) Photocatalytic activity of CdS (5 mg) loaded with 1 wt % of Pt, Au, Ru, Pd, MoS_2_, and 5 wt% FeP for 5 h under visible light irradiation. (**c**) FeP/CdS composite photocatalysts (1 mg) with different amounts of FeP over 5 h under visible light irradiation. (**d**) H_2_ evolution rates from a physical mixture of FeP and CdS (FeP + CdS) without grinding, CdS, FeP, and 1 mg of Fe_3_O_4_/CdS (with long term grinding) photocatalysts. Light resource: λ > 420 nm, LED: 30 × 3 W, 28 mW cm^−2^. Solution: lactic solution (10 mL, 10% v/v).

CdS has demonstrated that is an important photocatalyst for the photocatalytic HER because of its wide light-response range (with a direct bandgap of around 2.4 eV) and its high flat-band potential (−0.9 V vs. a normal hydrogen electrode)^9^,^35^,^36^,^37^. However, bare CdS is unstable during the photocatalytic reaction because of photocorrosion, where S^2−^ in CdS is oxidized by photogenerated holes accompanied with the elution of Cd^2+^ 38, which restricts its usefulness. To overcome this drawback, co-catalysts were generally loaded onto CdS to promote the rapid surface transfer of photogenerated electrons and holes from CdS^3^,^39^,^40^. Herein, in a typical experiment, we performed H_2_ evolution upon irradiation of a lactic acid solution (10% v/v, pH 2.0) containing the 5 wt% FeP/CdS photocatalysts (5 mg). The system continued to produce H_2_ at a constant rate for over 50 h (Fig. 2a), later the hydrogen evolution will become gently and there is still hydrogen evolution after 100 h irradiation. A control experiment with no FeP yielded no substantial H_2_ production (Fig. 2a). Cycle tests also demonstrated that the FeP/CdS composite photocatalysts had good stability after four cycles of photocatalysis under visible light irradiation (Fig. S5). This longevity may be attributed to the effective separation of photoexcited electrons and holes on CdS photocatalysts, decreasing the amount of photocorrosion. The decreasing rate of HER after 50 h of irradiation may be attributed to increasingly bare CdS photo-corrosion caused by the detachment of CdS and FeP. XRD analysis of 5 wt% FeP/CdS before and after photocatalysis for 100 h demonstrated that the crystallization of CdS clearly decreased after long periods of photocatalytic H_2_ evolution (Fig. S6).

To further verify the photocatalytic activity of FeP/CdS composite photocatalysts, some well-known co-catalysts such as MoS_2_, Pt, Pd, Ru, and Au were synthesized or *in situ* photo-deposited on the CdS surface and their activities were compared (in Fig. 2b). The results verified that compared to the other famous co-catalysts loaded with 1% on CdS, FeP/CdS shows the best H_2_ evolution activity, even 3 fold higher than *in situ* photo-deposited Pt-CdS photocatalysts. In addition, as we know, for Pt/CdS system, the hydrogen generation rate will clearly decrease after several hours irradiation because the noble Pt is easy poisoned by the – CO group from the degradation of lactic acid and lead to the deactivation of Pt^2^,^41^. However, FeP doesn’t display the poisoning phenomenon and FeP catalyst has demonstrated the excellent stability in strong acid solution^25^,^28^,^31^,^42^, which also supports the high activity and excellent stability of FeP.

Varying the amount of FeP in the present system clearly affected the photocatalytic activity of FeP/CdS composites. Figures 2c and S7 shows that 5 wt% FeP/CdS photocatalysts were the most active. The H_2_ evolution rate reached 202000 μmol h^−1^ g^−1^ after 5 h of irradiation, this corresponded to an apparent quantum efficiency (AQE) of over 35% upon excitation at 520 nm, more than 67 times the rate observed for pure CdS (3000 μmol h^−1^ g^−1^) which remained low because of the easy recombination of photoexcited electron-hole pairs. Evolution of H_2_ from the solution was very robust when using visible light irradiation (see supporting Movie 1). To the best of our knowledge, these are among the highest evolution activity and efficiency rates achieved over powdered photocatalysts for visible-light driven H_2_ production using non-noble metal, acid-stable HER catalysts (Table S1). The H_2_ evolution rates of 10 and 15 wt% FeP/CdS composite photocatalysts also showed rates of 180000 and 150000 μmol h^−1^ g^−1^, respectively. This trend indicates that after a peak FeP content the H_2_ evolution rate decreases, which may be attributed to overloading of black FeP particles would block the transition of photons^43^, in addition because the alone FeP can’t generate the H_2_, which is possible that the that FeP absorption in higher loaded samples *provides* parasitic absorption and could account for decreased performance.

Figure 2d shows the rate of H_2_ evolution from FeP/CdS, a physical mixture of FeP and CdS (FeP + CdS), CdS, FeP, and Fe_3_O_4_/CdS photocatalysts. The 5 wt% FeP/CdS photocatalysts exhibited the highest activity among the catalysts, with a rate that was more than 17 times greater than the rate for the physical mixture of 5 wt% FeP and CdS. This indicates that the intimate contact between FeP and CdS was crucial for the inter-electron transfer between the two components. Without FeP, CdS alone exhibited a very low H_2_ evolution rate, more than 67 times less than that of the 5 wt% FeP/CdS photocatalyst. Without photosensitizer CdS, FeP alone did not exhibit any photocatalytic activity. The 5 wt% Fe_3_O_4_/CdS exhibited activity levels that were slightly less than those for pure CdS, demonstrating that Fe_3_O_4_ is not a co-catalyst for H_2_ evolution in this CdS system. The control experiments results suggest that the strong and repeated grinding action effectively combined the materials and ensured the formation of a solid–solid interface between the FeP nanoparticles and the CdS support, contributing to the enhanced photocatalytic activity observed.

It was worth noting that the photocatalytic activity of the FeP/CdS system strongly depended on the pH of the lactic acid solution that acted as the proton source (Fig. S8). Therefore, we adjusted the pH value of solution using HCl and NaOH prior to irradiation to ensure the same concentration of protons from the lactic acid. The maximal H_2_ evolution rate was achieved at pH 2.0 although significant amounts of H_2_ were also obtained at both lower and higher pH values, similar to results observed in other systems^44^,^45^. This pH-dependency is related to the concentration of various ions at the catalyst surface, the dissociation equilibrium of lactic acid (HL ↔ H^+^ + L^−^), and the stability of CdS among other factors. For example, at higher pH values the unfavourable protonation of the reduced FeP will decrease the H_2_ evolution rate^44^. Conversely, at lower pH values the dissociation equilibrium is suppressed, decreasing the ability of lactic acid to function as a sacrificial electron donor, and the stability of CdS is decreased.

**Figure 3:**
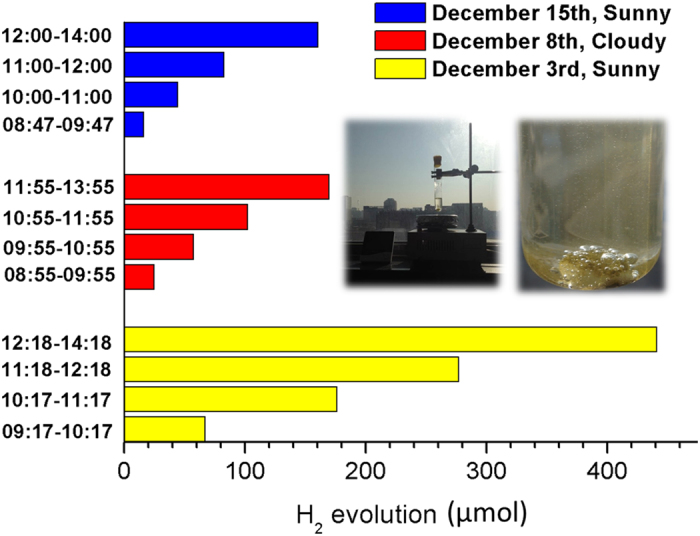
Photocatalytic activity under Sun light irradiation. Time courses of visible sola-light (glass filter) irradiation in laboratory using 5 wt% FeP/CdS photocatalyst (1 mg) at pH 2.0 in 10 mL H_2_O solution containing lactic acid (1 mL, 10% v/v). The light source was sunlight at December, 2014, in Beijing, China.

In order to further verify the activity of FeP/CdS photocatalysts, we also performed the photocatalytic hydrogen under irradiation by direct sunlight (Fig. 3). Here, a dramatic bubble was clearly generated when the quartz tube containing the catalysts and reactive solution was irradiated (as shown in the insert Figures). The robust photocatalytic activity of FeP/CdS photocatalyst was further demonstrated by a H_2_ evolution rate of 106000 μmol h^−1^ g^−1^ at midday on the first day of testing. In the natural sun irradiation movie in the air condition demonstrates also the continuous and robust hydrogen bubbles were generated using the composites catalysts (as shown in supporting Movie 2), which also shows the stability of catalysts under aerobic conditions.

### Photocatalytic mechanism

**Figure 4:**
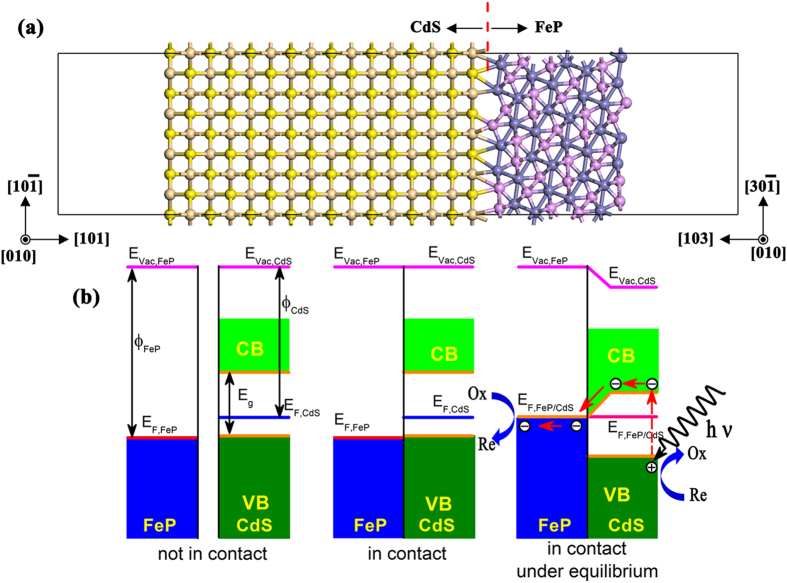
Schematic of photocatalysis by FeP/CdS composites. (**a**) The illustration of combination between metal FeP (P: lavender, Fe: purple) and semiconductor CdS (S: yellow, Cd: dull yellow). (**b**) Calculated energy band diagram of metal FeP and CdS.

To further explain the above experimental results, we carried out a preliminary density functional theory (DFT) calculation using the ultrasoft pseudopotential plane wave method implemented in the Cambridge Serial Total Energy Package (CASTEP) code^46^. The calculation model for the heterojunction metal-semiconductor structure of CdS (101)/FeP (103) is shown in Fig. 4a with the calculated energy band diagram illustrated in Fig. 4b. In the case of an isolated surface model (where FeP and CdS were not in contact with each other), the work functions of the metal FeP (103) and CdS (101) surfaces were 6.454 and 5.386 eV, respectively. Thus, when the heterojunction was formed by intense grinding, the contact of FeP and CdS creates an inherent electric field at the interface. This causes the energy band edges of CdS to be shifted downwards while the Fermi energy of FeP is shifted upwards (also known as band bending). After the heterojunction system reached equilibrium, the unified work function was observed to be 5.231 eV with the CB energy levels of CdS approximately equal to 3.57 eV, close to the experimental measurement of 3.49 eV^37^,^47^. Thus, the presence of the band bending can significantly depress the electron-hole pair recombination rate, consequently can promote more electrons to transfer out of the space-charge region at the interface to flow from the CdS layer to the FeP layer^48^,^49^. Also, the presence of the Schottky barrier means that the flow of electrons requires a larger amount of energy, which allows the photogenerated electron–hole pairs to be spatially separated by the FeP/CdS interface. Eventually, the oxidation reaction may occur on the CdS (101) surface with the separated holes, while the reduction reaction may occur on the FeP (103) surface with the separated electrons. In this way FeP loading can suppress the recombination of photogenerated electron–hole pairs, acting as an electron-acceptor in the composite photocatalyst system. Based on this theory, it is clear that H_2_ production by photocatalytic water splitting is possible, confirming the experimental observations above.

**Figure 5:**
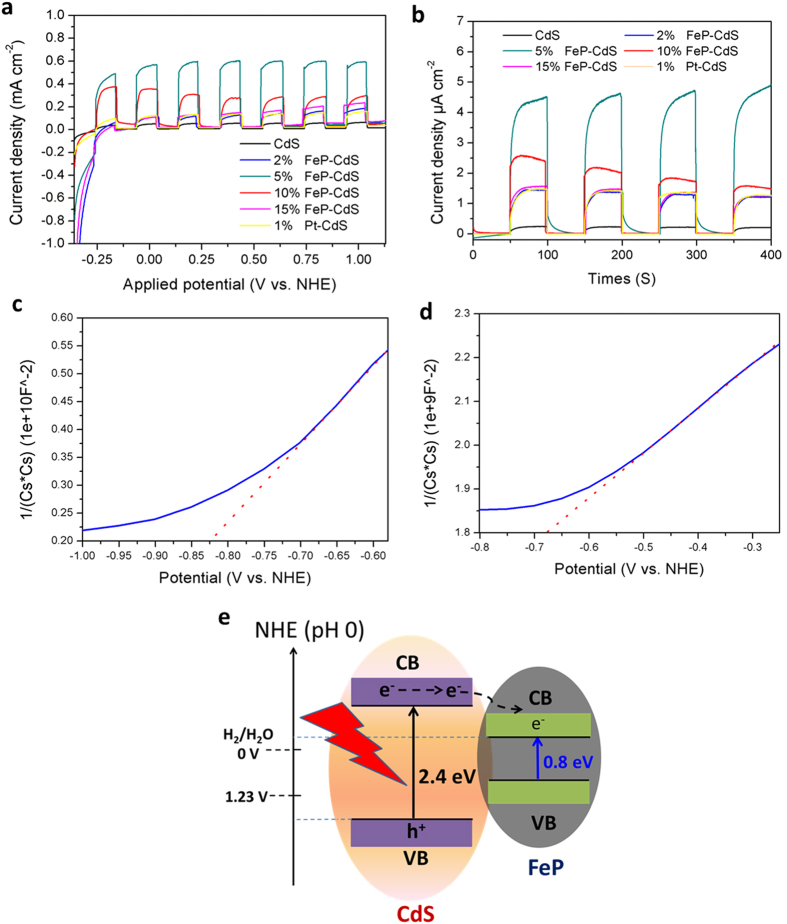
Photoelctrochemical characterization and Scheme of electron transfer. (**a**) I–V curve of various FeP co-catalysts and 1wt% Pt on CdS under visible irradiation in chopping mode in a 0.5 M Na_2_SO_4_ solution. (**b**) The unbiased On-Off photocurrent (I–t) of photoanodes including various FeP co-catalysts and 1 wt%Pt on CdS. A 300 W xenon arc lamp was used as the light source with a long-pass cut filter (λ > 420 nm). Mott–Schottky plot of (**c**) CdS nanoparticles and (**d**) FeP nanoparticles. (**e**) Energy level diagram to illustrate photocatalytic H_2_ evolution using FeP/CdS hybrid as a photocatalyst.

The photocurrent-voltage and the photocurrent-generated response experiments of FeP loaded onto CdS composites, were also used to verify that FeP served as an acceptor of CdS generated electrons and effectively suppressed charge recombination thereby lengthening the lifetime of the charge carriers (as shown in Fig. 5a,b). Figure 5a shows the I–V curve of the variously proportioned FeP co-catalysts in a 0.5 M Na_2_SO_4_ solution. All of the photocatalysts displayed the exhibited prompt andreproducible photocurrent, whereas without FeP, CdS generated a very low photoresponse. This indicates that loading FeP onto CdS can improve the charge transport from CdS to FeP, with the charge then passed onto the working electrode surface. The 5 wt% FeP loaded onto CdS exhibited the highest photocurrent density of all tested FeP photocatalysts, which is consistent with the photocatalytic activity results and again verifies that the 5 wt% FeP sample was the optimal content for photocatalytic H_2_ production. Importantly, we also compared the photoelectrochemical activity of 1 wt% Pt and 5 wt% FeP on CdS with the latter being found to have a much larger photocurrent density, indicating that FeP is excellent at assimilating electrons. Figure 5b shows the evolution of the photocurrent response over time with the 5 wt% FeP loaded on CdS, again displaying higher photocurrent densities than the other FeP co-catalysts and the 1wt% Pt on CdS.

As a typical “transition metal–metalloid” binary alloy, band gap theory also demonstrates through the Mott–Schottky curve^50^,^51^ that the conduction band of FeP is more negative than the energy level for H_2_ evolution (−0.059 V versus NHE, pH 1.0) and is more positive than the conduction band of CdS (as shown in Fig. 5c,d). Thus, the photogenerated electron from CdS can transfer to the conduction band of FeP, where it can be used in H_2_ production (Fig. 5e).

The work function, energy band gap theory, and photoelectrochemical results verified that FeP can effectively separate the photoexcited charge from CdS. It should be mentioned that not all long-lived charge carriers at the surface of FeP can contribute to the course of the photocatalytic reaction, because the active sites on surface of FeP will decide the surface reactions. Fortunately, much more active sites on the surface of FeP because of the binary alloy capability will further increase the hydrogen evolution reaction. Work focusing on the electrochemical H_2_ generation by transition metal phosphides has demonstrated that both the metal centre (Fe) and the pendant base (P) are active sites for H_2_ production^22^,^23^,^28^,^31^,^52^. We performed the x-ray photoelectron spectroscopy (XPS) results showed that Fe and P in FeP NPs carry a partial positive charge (δ^+^) and a partial negative charge (δ^−^), respectively (Fig. S9). This implies electron transfer from Fe to P in FeP^28^,^53^,^54^. Metal Fe exhibits strong binding to H and acts as the hydride acceptor. Meanwhile, P sites play a crucial role by acting as a proton acceptor or the delivery site for H, allowing P to facilitate the formation of iron hydride to be used in subsequent H_2_ evolution^55^. This is similar to the situation observed with the [FeFe] or [NiFe] hydrogenase where the active sites feature pendant bases proximate to their metal centres. Metal-complex HER catalysts also incorporate proton relays from pendant acid/base groups positioned close to the metal centre where H_2_ evolution occurs^56^,^57^.

## Discussion

As a typical “transition metal-metalloid” binary alloy, FeP displayed some capabilities common among alloys. For example, a number of electrochemical analyses confirmed that both the metal centre (Fe) and the pendant base (P) are active sites for H_2_ generation. The FeP nanoparticles also combined the merits of both metallic nanoparticles and metal complexes while simultaneously avoiding the decomposition associated with molecular catalysts, which together enabled highly efficient and robust photocatalytic H_2_-production. However, another band gap theory that illustrates how FeP functions as a semiconductor remained outstanding catalytic activity. In order to further study the properties, we also calculated the band structure of FeP (Figure S10). In the band structure of FeP, one can see that the highest occupied states and the lowest unoccupied states are contacted together near the k-point of G/S and the k-line of U-R, implying the zero band gap (i.e. presents conducting feature) along these wave vector directions. On the other hand, on the k-lines of Z-T and T-Y, there is an obvious band gap (about 1 eV), indicating that the semiconducting feature. This calculated result shown the anisotropy property for electronic structure of FeP, resulting in the semiconducting feature. Furthermore, in the partial density of states of FeP, one can see that Fe-3d states continuously fill the energy region near the E_F_ level, which is the origin of the conducting feature of FeP; while above/below the E_F_ level, there are hybridized states between Fe-3d states and P-3p states, which is the origin of the semi-conducting feature of FeP. Therefore, we combined the experimental analysis and DFT calculations both from the alloy and the semiconductor to demonstrate the mechanism that explains how using FeP as a co-catalyst enhanced the H_2_ generation activity of CdS.

In summary, a low-cost yet highly efficient and stable semiconductor FeP/CdS photocatalytic system was successfully established using lactic acid as the electron donor. The robust photocatalytic activity was demonstrated by an especially high H_2_ evolution rate of 202000 μmol h^−1^ g^−1^ for a FeP/CdS sample during 5 h of visible lightirradiation, and the activity showed an AQE of over 35% at 520 nm, considerably better than that of more common co-catalysts—such as Pt, Ru, and MoS_2_,—and 3-fold higher than that of the control *in situ* photodepositedPt/CdS system under the same experimental conditions. The system also performed well under solar irradiation with a rate of 106000 μmol h^−1^ g^−1^ at midday on the first day of testing. More importantly, FeP/CdS photocatalysts showed impressive photochemical stability even after 100 h of irradiation. The essential thermodynamic relationship and the mechanism of effective charge separation based on the band alignment between the CdS and FeP was elucidated, which will be useful in providing insight for the design and preparation of efficient semiconductor-based, water-based, artificial H_2_-generating photocatalysts that function under visible light irradiation.

## Methods

### Preparation of Fe_3_O_4_ NPs

All materials were of analytical grade and used as received without further purification. Fe_3_O_4_ NPs were prepared using the modified hydrothermal method reported by Li *et al.*^58^ (see Figs S1–S3). Principally, 1.35 g of FeCl_3_·6H_2_O was added to 25 mL of ethylene glycol. The solution was vigorously stirred until it became transparent. Then, 2.70 g of sodium acetate and 1.0 g of sodium citrate were dissolved into the solution while stirring. The transparent solution was poured into a 50 mL Teflon-lined autoclave and heated at 200 °C for 10 h. The product was then centrifuged and washed several times with deionized water and ethanol. The product was then dried at 80 °C for 12 h in a vacuum oven. The black powder obtained was Fe_3_O_4_ NPs.

### Preparation of FeP NPs

Fe_3_O_4_ (100 mg) and NaH_2_PO_2_ (500 mg) were ground using an agate mortar. The mixture was then transferred into a porcelain boat and calcined in a tubular furnace under a flow of Ar gas for 3 h with a heating rate of 2 °C min^−1^ up to 400 °C to obtain the FeP NPs. The NPs were washed with deionized water and ethanol several times and then dried in a vacuum oven at 40 °C for 8 h to yield the black, solid FeP NPs.

### Synthesis of the CdS NPs

In a typical synthesis, Na_2_S solution was added dropwise into a CdCl_2_ solution to yield a molar ratio of Cd:S of 1:1.2. The mixed solution was strongly stirred for 24 h and then left to stand for another 24 h. The product was centrifuged and washed with deionized water several times before undergoing ultrasonic treatment with water and being put into a 100 mL Teflon-lined autoclave. The product was then heated at 200 °C for 24 h, filtered, and washed by deionized water and ethanol several times before being dried at 80 °C for 10 h to obtain the CdS NPs.

Various proportions of FeP and CdS NPs were combined by strongly grinding them together in the agate mortar for a long period (40 min) to ensure the formation of a robust solid–solid interface between the FeP NPs and the CdS supports. MoS_2_ NPs and MoS_2_-CdS composites were synthesized by modifying the existing literature^2^. Typically, a mixture of 0.25 g of Na_2_MoO_4_ and 0.2 g of L-cysteine was dissolved in 40 mL of deionized water which was then transferred into a 50 mL Teflon-lined stainless steel autoclave and heated at 180 °C for 24 h. After cooling naturally, the precipitates were collected by centrifuge, washed, and dried in a vacuum oven. To prepare the 1% MoS_2_-CdS composite, CdCl_2_ and as-prepared MoS_2_ were dispersed in water with polyvinylpyrrolidone under an Ar atmosphere. Thioglycolic acid was then added to the solution which was stirred for 2 h before 0.05 M Na_2_S·9H_2_O was added. This mixture was then continuously stirred for 2 days. Finally, the products were washed and annealed at 300 °C for 2 h in an Ar atmosphere.

Noble metals including Pt, Ru, Pd, and Au were loaded on CdS using *in situ* the photoreduction method with aqueous solutions of H_2_PtCl_6_, RuCl_3_, PdCl_2_, and HAuCl_4_, respectively.

### Sample characterization

The crystalline structures of the samples were determined by XRD (Bruker D8 Focus) with Cu-K_α_ radiation (λ = 1.54056 Å). The morphologies were obtained from a TEM (JEM 2100F) that was operated at an accelerating voltage of 200 kV. The scanning electron microscope (SEM) images and the energy dispersive X-ray spectrometry (EDX) analyses were carried out by a field emission SEM (S-4800, Hitachi) operating at 5 kV. XPS data were obtained with an electron spectrometer (ESCALab220i-XL, VG Scientific) using 300 W Al Kα radiation. The base pressure was approximately 3 × 10^−9^ mbar. The binding energies were referenced to the C1s line at 284.8 eV from adventitious carbon. The UV-vis absorption spectrum was investigated on a spectrophotometer (U-3010, Hitachi).

### Photocatalytic H_2_ generation

H_2_ production was carried out in a 50 mL quartz cuvette containing 1 mg of FeP/CdS photocatalyst in a 10 mL aqueous solution containing lactic acid (1 mL, 10% v/v) in a quartz cuvette reaction cell. The cuvette was sealed with a rubber septum and degassed by bubbling Ar through the solution for 40 min at atmospheric pressure. Then, the mixture was irradiated using LEDs (λ > 420 nm, LED: 30 × 3 W, 16 mW/cm^2^) while stirring. All of the experiments were conducted at room temperature with distilled water. A 0.6 mL gas was intermittently sampled through the septum and was analyzed by a TCD for the quantification of H_2_ using a gas chromatograph (GC-14C, Shimadzu Co.) equipped with a column (3 m × 2 mm) of 5 Å molecular sieves, a thermal conductivity detector, and Ar as the carrier gas. The amount of H_2_ evolved was calculated relative to the amount of photocatalyst in the system. White LED light source (30 × 3W, λ > 420 nm) were used as the irradiation light sources.

The AQE was measured by the similar method, just applying a Xe lamp (300 W) with a 520 nm bandpass filter (MIF-W, Ceaulight Co., China) as the irradiation light. The number of incident photons was measured using a radiant power energy meter. The total intensity of irradiation was estimated by averaging 20 points of the irradiation area. The AQE was calculated using the following equation:


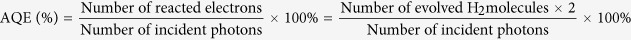


The film electrodes of different photocatalysts for the photoelectrochemical response measurements were firstly fabricated. The powders and ethanol were mixed homogeneously (150 mg mL^−1^), and the obtained paste was then spread on the conducting fluorine-doped SnO_2_ glass substrate (FTO, 15 U per square) with a glass rod, using adhesive tapes as spacers. The resulting films have ca. 4.0 mm thickness and 1.0 cm^2^ active area.

Photoelectrochemical activity measurements were performed with a CHI electrochemical analyser (Chenhua electrochemical workstation) in a standard three-electrode system using the prepared samples as the working electrodes with an active area of approximately 1.0 cm^2^, a Pt sheet as the counter electrode, and a saturated calomel electrode (SCE) as a reference electrode. A 300 W Xe lamp with a monochromator and a cutoff filter was used as the light source.

### DFT calculations

The DFT calculations were carried out by the Cambridge Serial Total Energy Package (CASTEP) codes. The ultrasoft pseudopotential was chosen to deal with the interaction between the ion core and valence electrons. The exchange and correlation effects among valence electrons were described by the Perdew–Burke–Ernzerhof version of the generalized gradient approximation. The Kohn–Sham wave functions of the valence electrons were expanded using a plane-wave basis set within a specified energy cutoff chosen at 340 eV. Using the periodic slab model and self-consistent dipole correction, the averaging electrostatic potential in the planes perpendicular to the slab normal could be obtained. Thus, the change in electrostatic potential through the slab could be plotted. Furthermore, the plot of electrostatic potential also contained the value of work function calculated as the difference between the potential level in a vacuum and the Fermi energy.

## Additional Information

**How to cite this article**: Cheng, H. *et al.* Robustly photogenerating H_2_ in water using FeP/CdS catalyst under solar irradiation. *Sci. Rep.* **6**, 19846; doi: 10.1038/srep19846 (2016).

## Supplementary Material

Supporting Movie 1

Supporting Movie 2

Supplementary Information
