# Supplementary material for: Robustly photogenerating H2 in water using FeP/CdS catalyst under solar irradiation
Source: Sci Rep. 2016 Jan 28;6:19846. doi: 10.1038/srep19846 (PMC4730145; doi:10.1038/srep19846)
Supplement: Supplementary Information [file srep19846-s3.doc]

Supporting Information

Robustly photogenerating H_2_ in water using FeP/CdS catalyst under solar irradiation

Huanqing Cheng,^1,2^ Xiao-Jun Lv,^1*^ Shuang Cao,^1^ Zong-Yan Zhao,^3^ Yong Chen,^1^ Wen-Fu Fu^1,2*^

1. Key Laboratory of Photochemical Conversion and Optoelectronic Materials and HKU-CAS Joint Laboratory on New Materials, Technical Institute of Physics and Chemistry, University of Chinese Academy of Sciences, Chinese Academy of Sciences, Beijing 100190, P. R. China. E-mail: [xjlv@mail.ipc.ac.cn](mailto:xjlv@mail.ipc.ac.cn), fuwf@mail.ipc.ac.cn
2. College of Chemistry and Chemical Engineering, Yunnan Normal University, Kunming 650092, P. R. China
3. Faculty of Materials Science and Engineering, Kunming University of Science and Technology, Kunming 650093, People’s Republic of China.


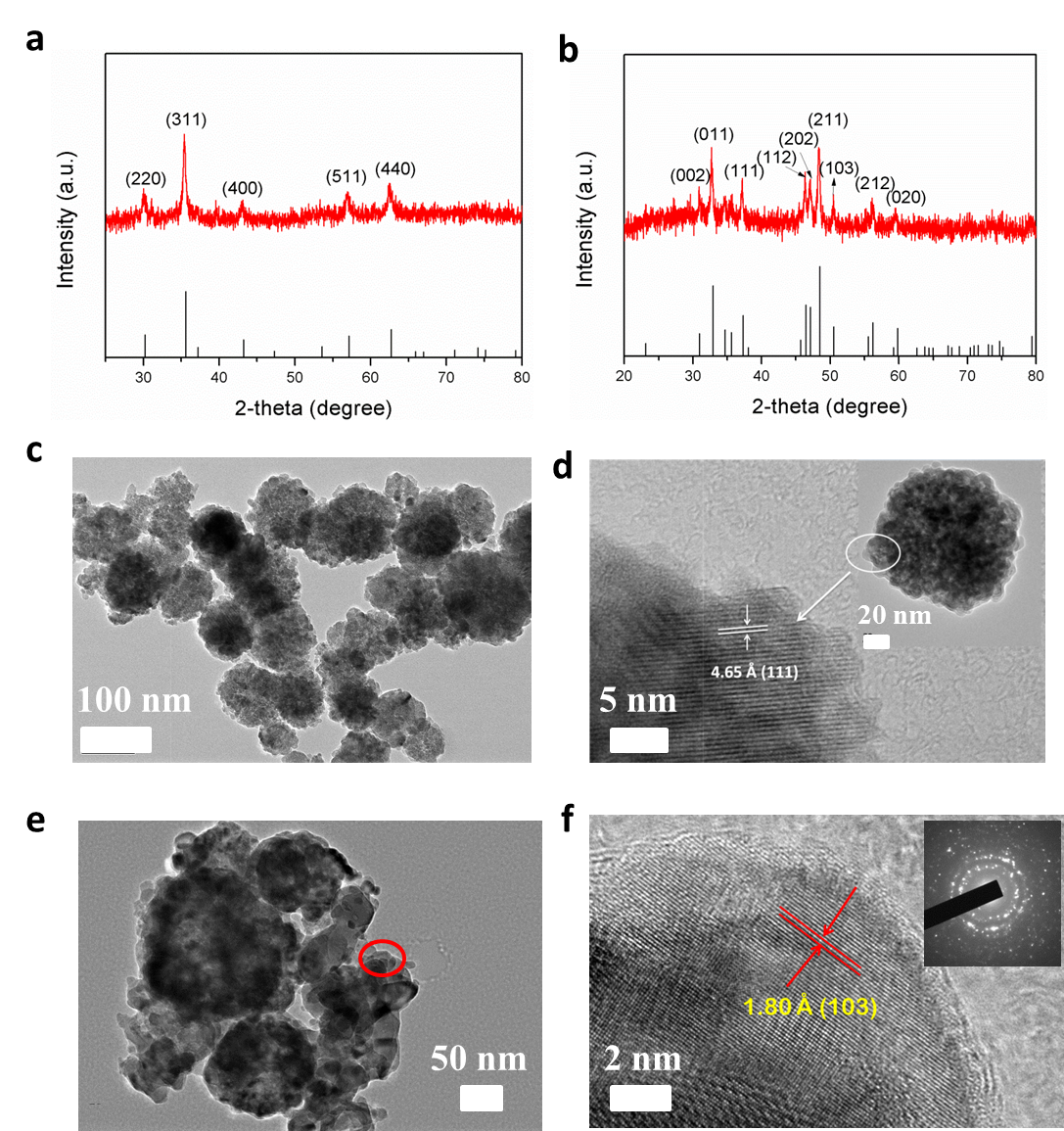


**Figure S1.** **Characterization of Fe_3_O_4_ and FeP.** XRD patterns for (**a**) Fe_3_O_4_ nanoparticles and (**b**) FeP nanoparticles. (**c**) TEM images of Fe_3_O_4_ nanoparticles. (**d**) HRTEM image of Fe_3_O_4_ nanoparticles taken from the area marked with a white circle. (**e**) TEM image of FeP nanoparticles. (**f**) HRTEM image taken from the area marked with a red circle in (**e**).

Fig. S1a shows the X-ray diffraction patterns of Fe_3_O_4_ NPs and the diffraction peaks characteristic of JCPDS 75-1609.^1^ Fig. S1b shows the X-ray diffraction patterns for the phosphidated product and the peaks corresponding well to the MnP-type FeP (JCPDS No. 78-1443),^2,3^ which confirms the formation in high yield of FeP NPs. Fig. S1 c show transmission electron microscopy (TEM) images of Fe_3_O_4_ NPs and the size of Fe_3_O_4_ nanoparticle is 100~200 nm. A high-resolution TEM (HRTEM) image of the selected area in Fig. S1d shows well-resolved lattice fringes with distances of 4.65 Å, corresponding to the (111) plane of Fe_3_O_4_. Fig. S1e shows TEM images of FeP NPs and confirmed the preservation of the nanoparticles morphology in the Fe_3_O_4_ precursor after phosphidation. In addition, the size of FeP is almost coincident with the Fe_3_O_4_ precursor. A HRTEM image of the selected area in Fig. S1f reveals the lattice fringes with interplanar distances of 1.8 Å, which corresponded well to the (103) planes of FeP. The corresponding selected area electron diffraction (SAED) pattern exhibits discrete spots of FeP.


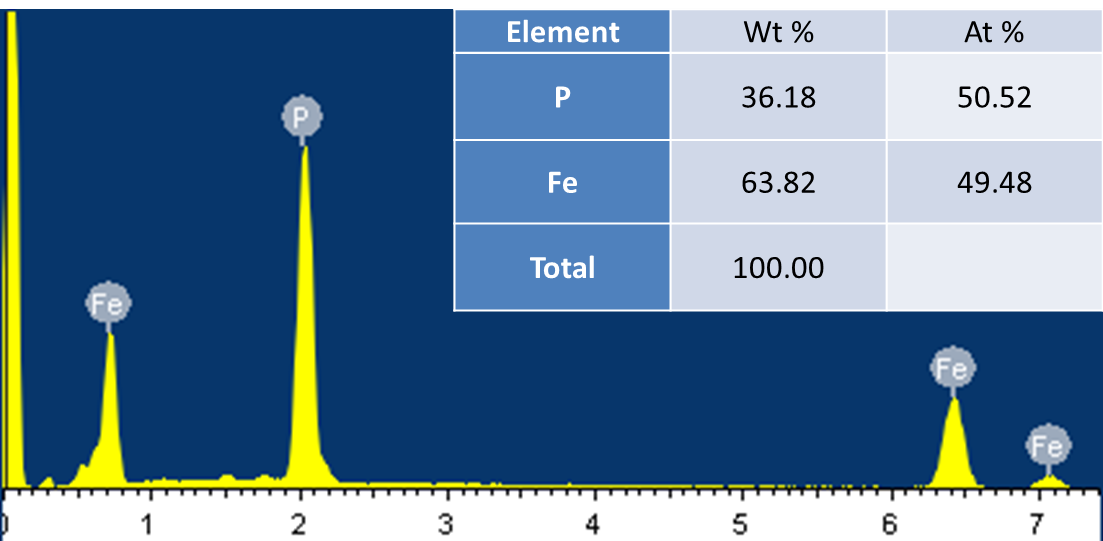


**Figure S2.** EDX spectrum for FeP NA/Ti.

The energy dispersive X-ray (EDX) spectrum (Fig. S2) indicates a 49.48:50.52 Fe:P ratio in the FeP nanoparticles, consistent within experimental error with the expected 1:1 FeP stoichiometry.


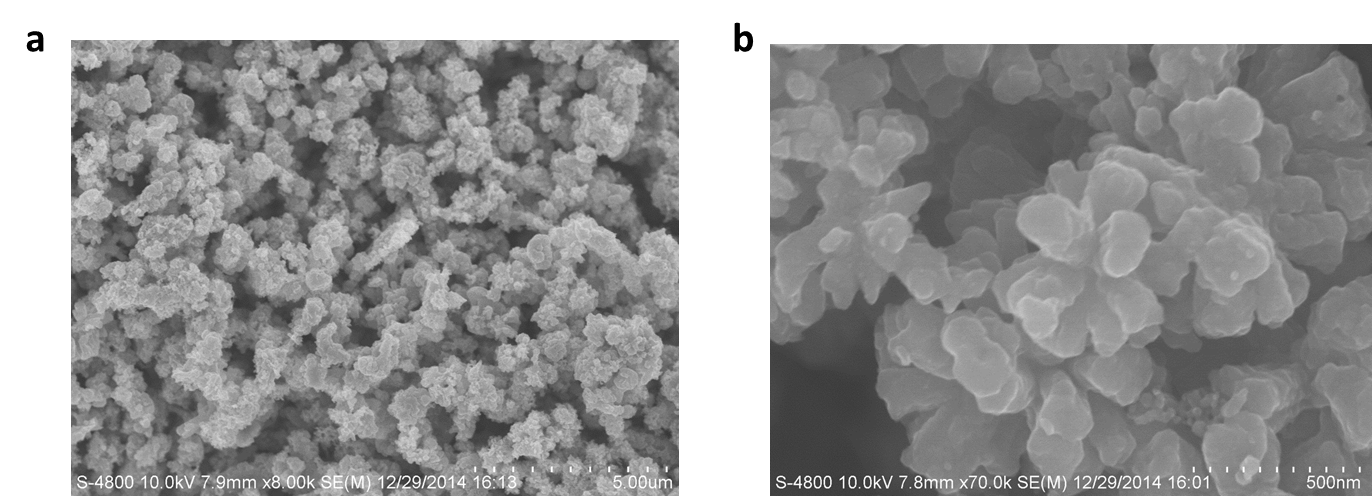


**Figure S3.** SEM images of the as-prepared FeP.


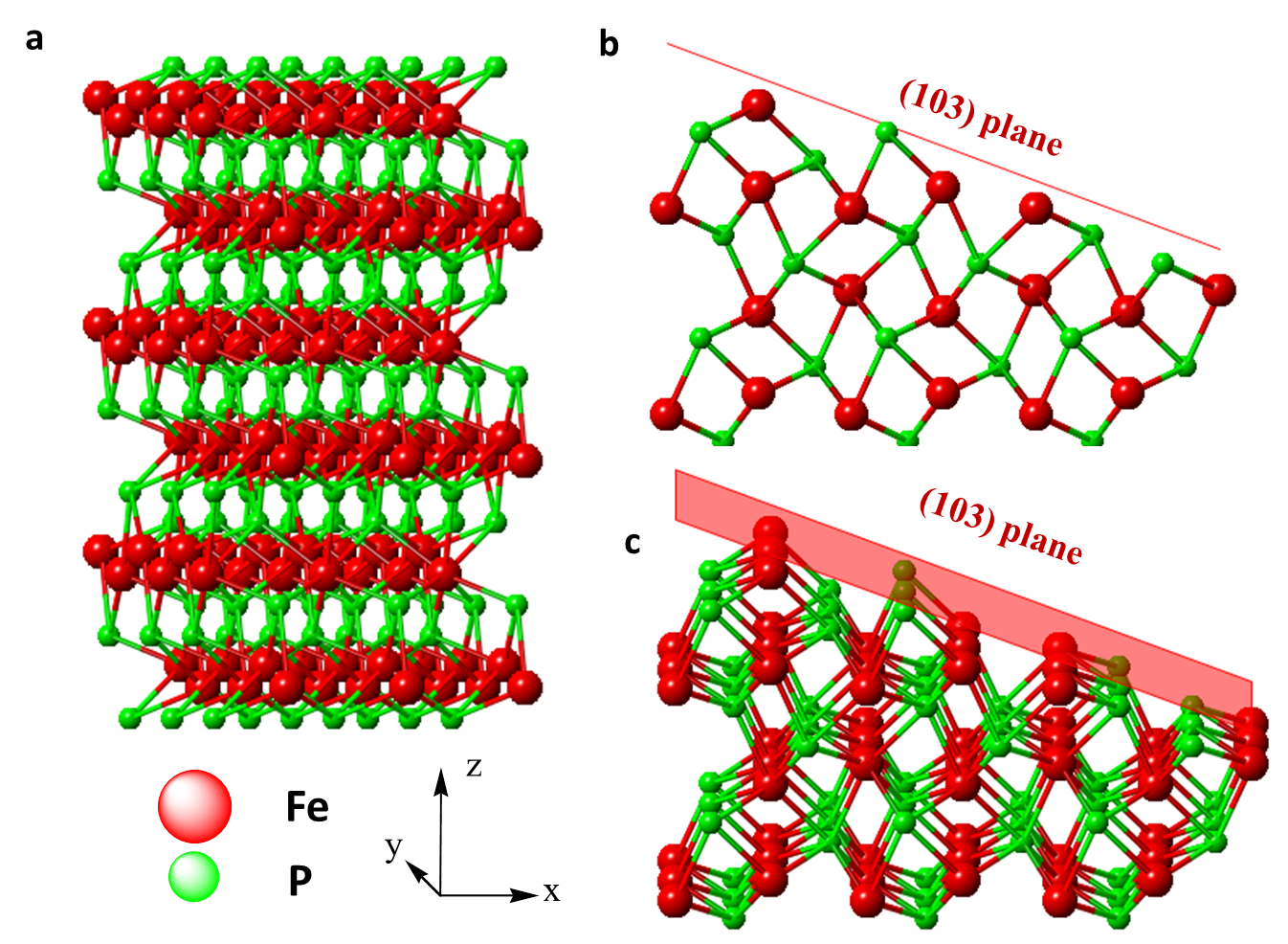


**Figure S4.** **Crystal structure of FeP.** (**a**) three unit cells stacked on top of one another, with a single unit cell outlined, (**b**) the structure of the FeP (103) surface, and (**c**) a two-dimensional slice of FeP, showing the (103) surface on top.


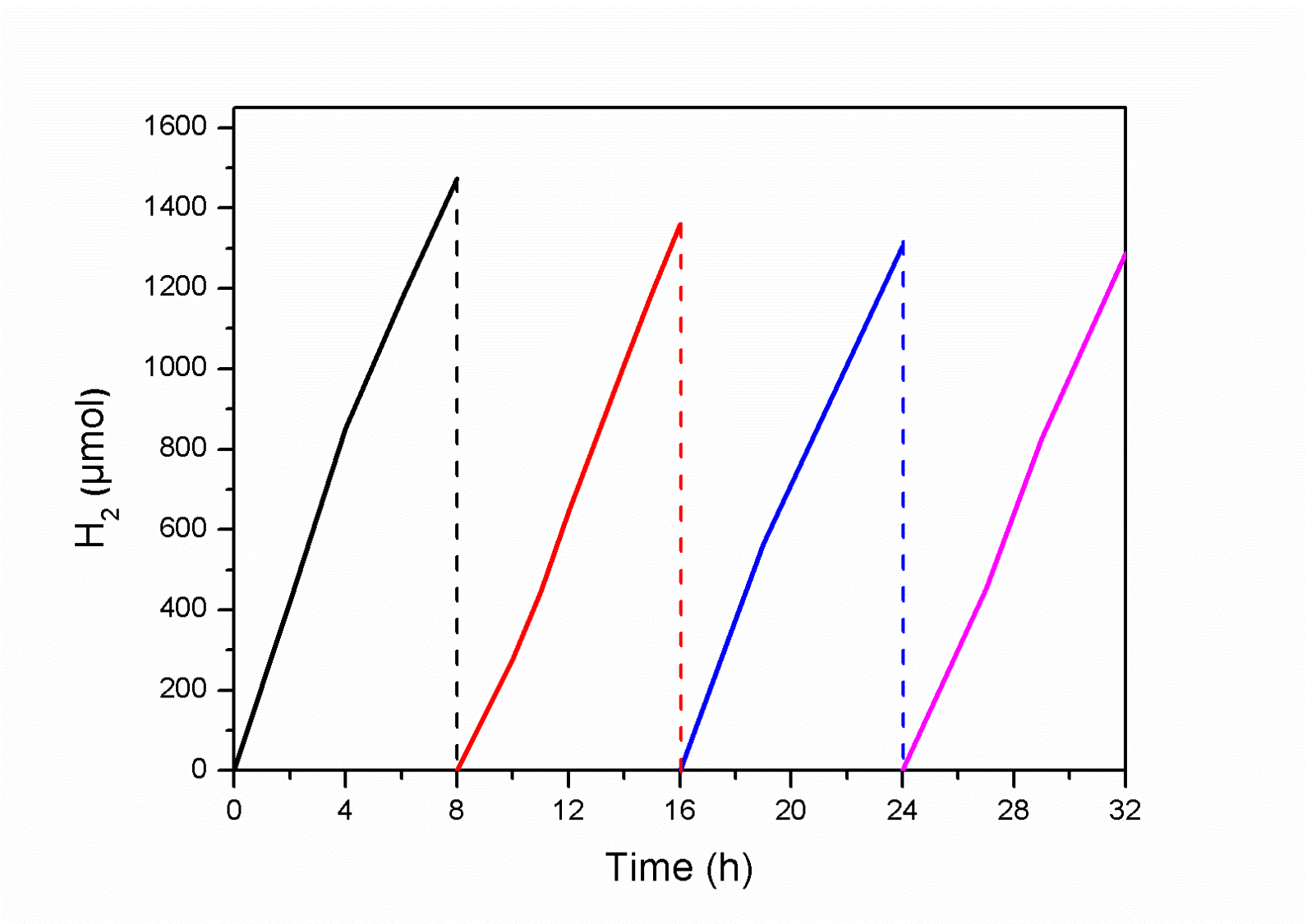


**Figure S5.** **Photocatalytic activity.** Hydrogen generation capability of 5% FeP/CdS photocatalysts (1mg) under visible light (≥ 420 nm, LED: 30 × 3 W) irradiation in 10 mL H_2_O solution containing lactic acid (1 mL; 10%, v/v).


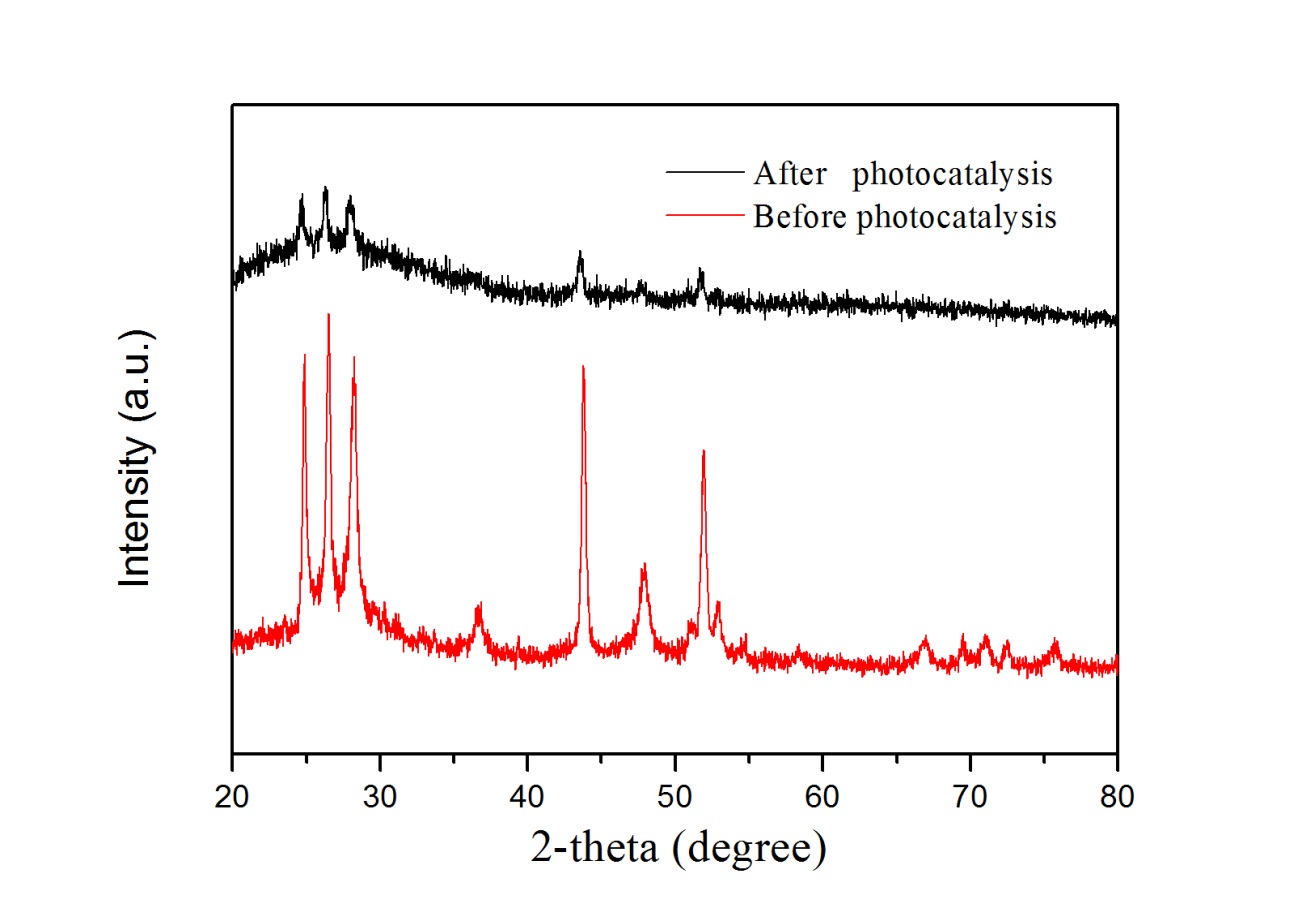


**Figure S6.** The XRD of 5% FeP-CdS composites photocatalyst before and after photocatalysis for 100 h.


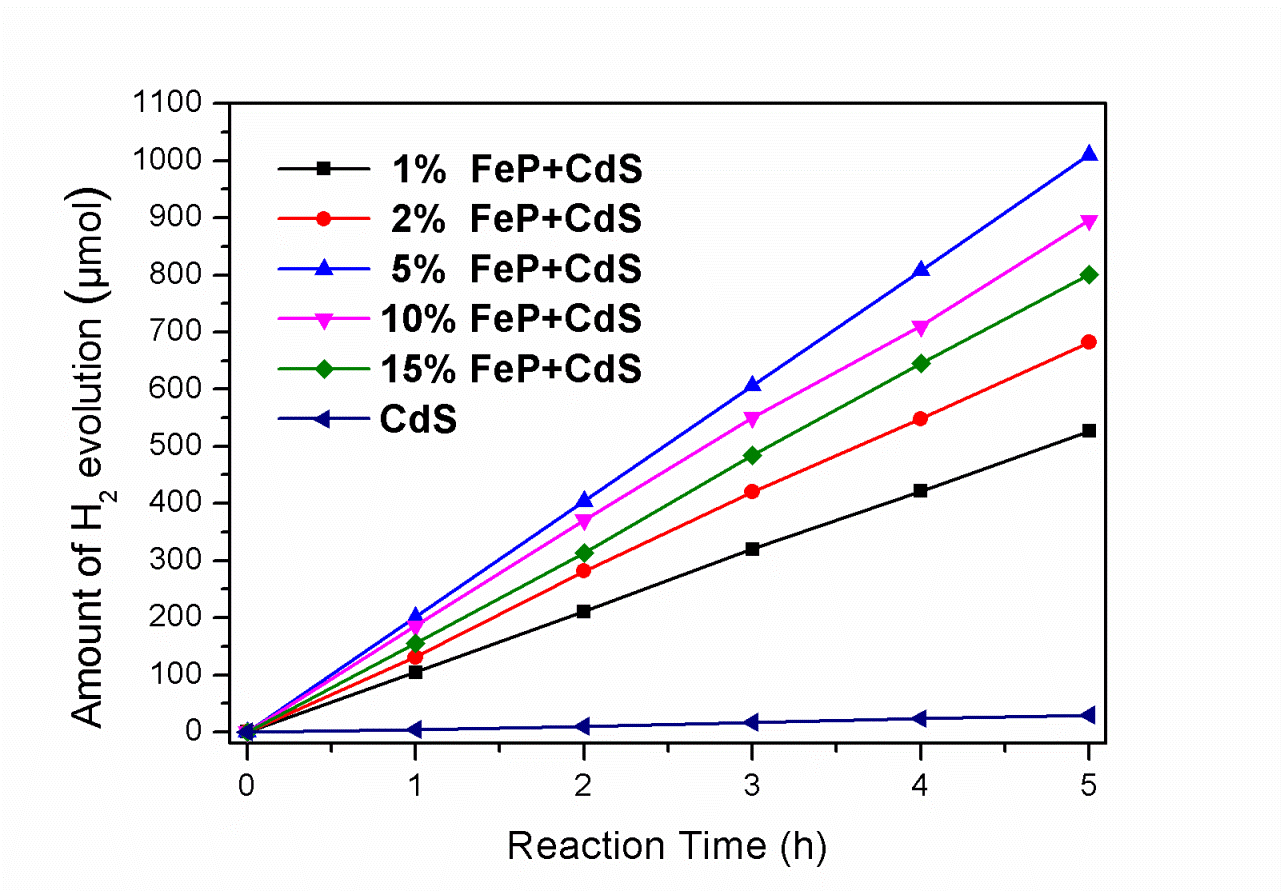


**Figure S7.** **Photocatalytic activity.** FeP/CdS composite photocatalysts (1 mg) with different amounts of FeP over 5 h under visible light irradiation.


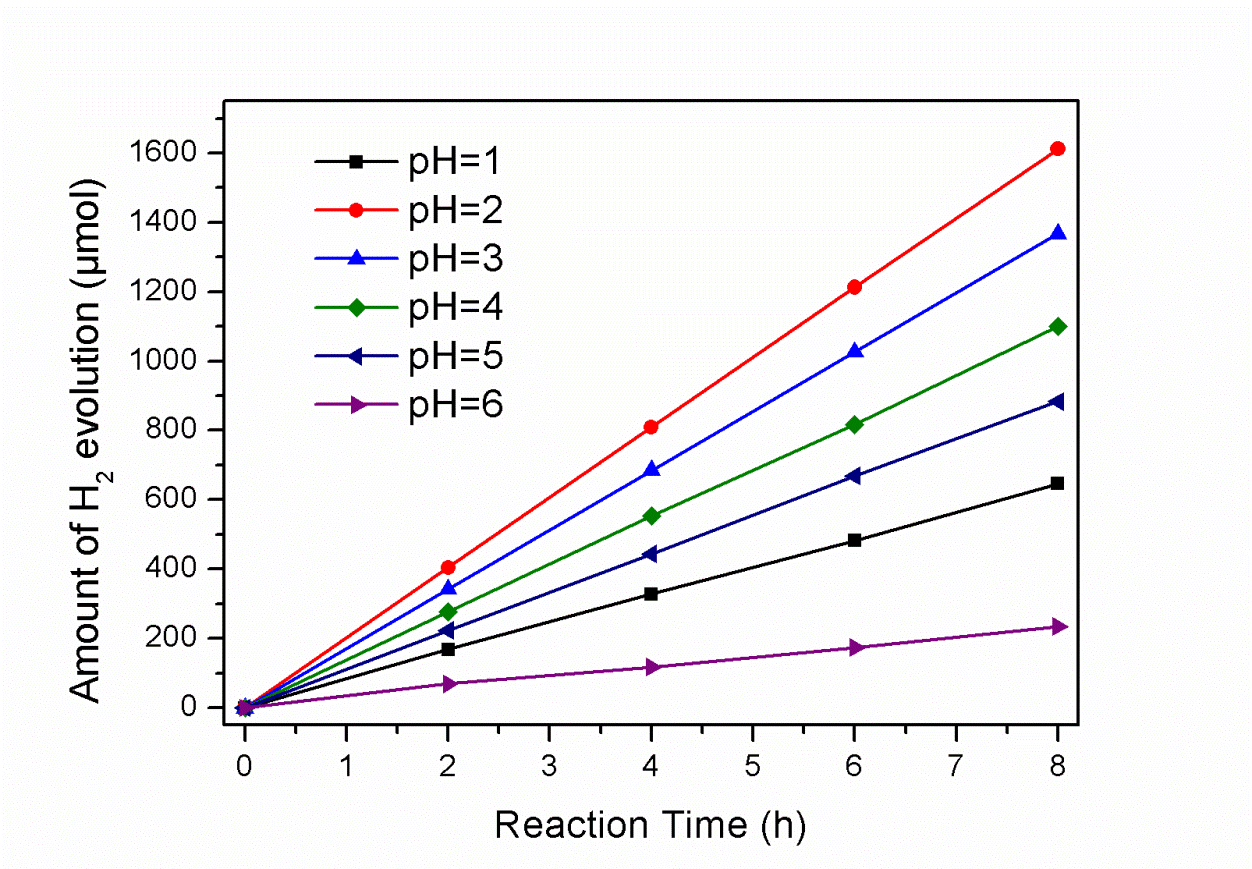


**Figure S8.** **Photocatalytic activity.** H_2_ evolution over time from visible light (λ > 420 nm, LED: 30 × 3 W) irradiation of 5 wt% FeP/CdS photocatalyst (1 mg) at different pH values in 10 mL H_2_O solution containing lactic acid (1 mL, 10% v/v).


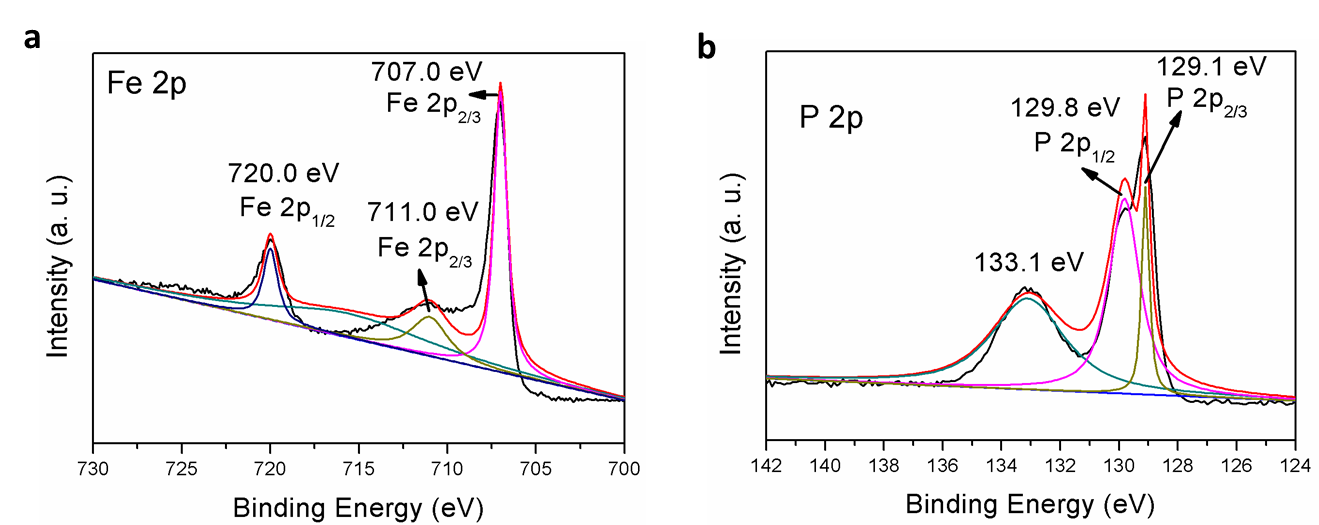


**Figue** **S9.** XPS spectra in the (**a**) Fe (2p) and (**b**) P (2p) regions for FeP.

Fig. 9 shows the X-ray photoelectron spectroscopy (XPS) 2p spectra in the Fe(2p) and P(2p) regions of FeP. The peaks for the binding energy of Fe 2p_3/2_ appear at 707 and 711 eV, and the peak at 720 eV is the binding energy of Fe 2p_1/2_. The P 2p spectrum shows two peaks at 129.1 and 129.8 eV corresponding to the BE of P 2p1/2 and P 2p3/2, respectively. In addition, the spectrum of P 2p also exhibits a peak with high BE of 133.1 eV, which is consistent with the previous reports and could be attributed to PO_4_^3-^ or P_2_O_5_ caused by oxidation due to air contact along with one peak at 133.1 eV.^3-5^ Among the peaks for the binding energy of Fe 2p_3/2_ appear at 707 and P 2p1/2 at 129.1 eV are close to the BE for Fe and P in FeP.^6^ Furthermore, Sun et al reported that the Fe 2p BE of 707.0 eV is positively shifted from that of metallic Fe (706.8 eV) while the P 2p BE of 129.3 eV shows negative shift from that of elemental P (130.2 eV), suggesting Fe and P in FeP NWs carry partial positive charge (d^+^) and negative charge (d^-^), respectively. The results imply the occurrence of electron transfer from Fe to P in FeP.^3,6,7^


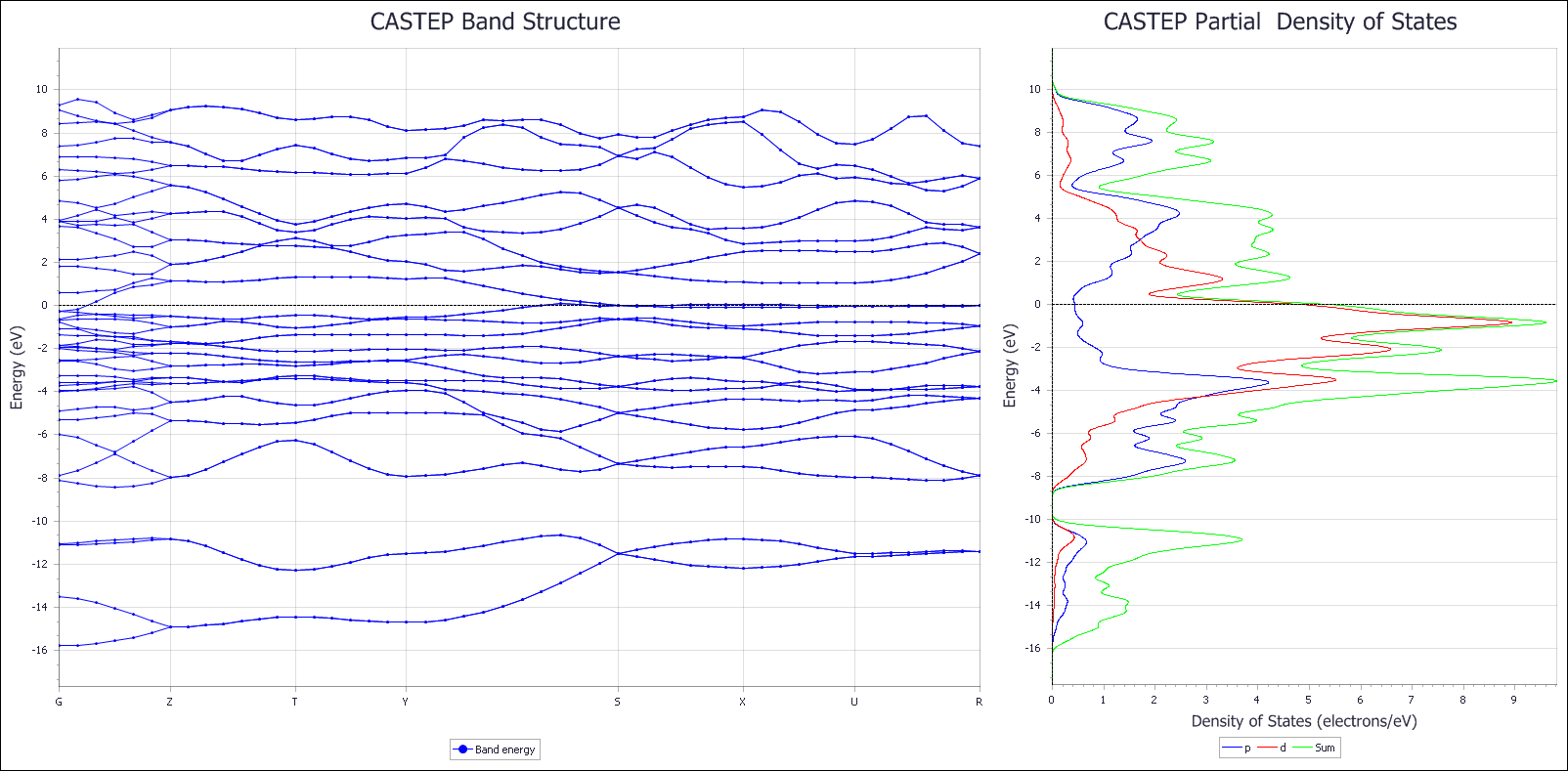


**Figure S10.** The calculated band structure of FeP.

**Table S1 Comparison of HER performance in acidic media for FeP cocatalyst with other cocatalysts on CdS.**

| **Photocatalysts** | **HER rate [μmol h^-1^ g^-1^]** | **AQY [%]** | **Different sacrificial agent** | **Irradiation light source** | **Ref.** |
| --- | --- | --- | --- | --- | --- |
| 2% MoS_2_-Graphene/  CdS | 9000 | 28.1 (420 nm) | Lactic acid | 300W xenon lamp (*λ* > 420 nm) | 8 |
| 0.2% MoS_2_/CdS | 5400 | N/A | Lactic acid | 300W xenon lamp (*λ* > 420 nm) | 9 |
| (0.5 % Pt + 1.12% Gr)Pt-Graphene/CdS | 56000 | 22.5 (420 nm) | Lactic acid | 350 W xenon arc lamp (≥420 nm) | 10 |
| 1% Pt-PdS/CdS | 29200 | 93 (420 nm) | Na_2_S-Na_2_SO_3_ | 300W xenon lamp (*λ* > 420 nm) | 11 |
| 1% Pd/CdS | 12700 | 40 (420 nm) | Na_2_S-Na_2_SO_3_ | 300W xenon lamp (*λ* > 420 nm) | 11 |
| 1% Pt/CdS | 16000 | 51 (420 nm) | Na_2_S-Na_2_SO_3_ | 300W xenon lamp (*λ* > 420 nm) | 11 |
| 1% Pt-Pd/CdS | 16500 | 53 (420 nm) | Na_2_S-Na_2_SO_3_ | 300W xenon lamp (*λ* > 420 nm) | 11 |
| 1% PdS/CdS | 20000 | 34 (420 nm) | Na_2_S-Na_2_SO_3_ | 300W xenon lamp (*λ* > 420 nm) | 11 |
| CdS-ZnS  core-shell | 800 | N/A | Na_2_S-Na_2_SO_3_ | 300 W Xe lamp (*λ* > 400 nm) | 12 |
| 1 % Ni(OH)_2_-CdS/rGO | 4730 | N/A | Na_2_S-Na_2_SO_3_ | 300W xenon lamp (*λ* > 420 nm) | 13 |
| 1.2 mol% NiS/CdS | 7300 | 51.3 (420 nm) | Lactic acid | 300W xenon lamp (*λ* > 420 nm) | 14 |
| 5% FeP/CdS | 206000 | 35 (520 nm) | Lactic acid | 3 W LED lamp (*λ* > 420 nm) | This work |
| 5% FeP/CdS  (solar light) | 106000 | N/A | Lactic acid | 3 W LED lamp (*λ* > 420 nm) | This work |

**Reference**

1 Deng, H. *et al.* Monodisperse Magnetic Single-Crystal Ferrite Microspheres. *Angew. Chem. Int. Ed.* **44**, 2782–2785 (2005).

2 Callejas, J. F. *et al.* Electrocatalytic and Photocatalytic Hydrogen Production from Acidic and Neutral-pH Aqueous Solutions Using Iron Phosphide Nanoparticles. *ACS Nano* **8**, 11101–11107 (2014).

3 Jiang, P. *et al.* A Cost-Effective 3D Hydrogen Evolution Cathode with High Catalytic Activity: FeP Nanowire Array as the Active Phase. *Angew. Chem. Int. Ed.* **53**, 12855–12859 (2014).

4 Zhang, Z., Lu, B., Hao, J., Yang, W. & Tang, J. FeP nanoparticles grown on graphene sheets as highly active non-precious-metal electrocatalysts for hydrogen evolution reaction. *Chem. Commun.* **50**, 11554–11557 (2014).

5 Bai, J., Li, X., Wang, A., Prins, R. & Wang, Y. Hydrodesulfurization of dibenzothiophene and its hydrogenated intermediates over bulk MoP. *J. Catal.* **287**, 161–169 (2012).

6 Grosvenor, A. P., Wik, S. D., Cavell, R. G. & Mar, A. Examination of the Bonding in Binary Transition-Metal Monophosphides MP (M = Cr, Mn, Fe, Co) by X-Ray Photoelectron Spectroscopy. *Inorg. Chem.* **44**, 8988–8998 (2005).

7 Li, L., Chen, C., Chen, L., Zhu, Z. & Hu, J. Catalytic Decomposition of Toxic Chemicals Over Iron Group Metals Supported on Carbon Nanotubes. *Environ Sci. Technol.* **48**, 3372–3377 (2014).

8 Chang, K. *et al.* MoS_2_/Graphene Cocatalyst for Efficient Photocatalytic H2 Evolution under Visible Light Irradiation. *ACS Nano* **8**, 7078–7087 (2014).

9 Zong, X. *et al.* Enhancement of photocatalytic H2 evolution on CdS by loading MoS2 as cocatalyst under visible light irradiation. *J. Am. Chem. Soc.* **130**, 7176–7177 (2008).

10 Li, Q. *et al.* Highly Efficient Visible-Light-Driven Photocatalytic Hydrogen Production of CdS-Cluster-Decorated Graphene Nanosheets. *J. Am. Chem. Soc.* **133**, 10878–10884 (2011).

11 Yan, H. *et al.* Visible-light-driven hydrogen production with extremely high quantum efficiency on Pt–PdS/CdS photocatalyst. *J. Catal.* **266**, 165–168 (2009).

12 Xie, Y. P., Yu, Z. B., Liu, G., Ma, X. L. & Cheng, H.-M. CdS–mesoporous ZnS core–shell particles for efficient and stable photocatalytic hydrogen evolution under visible light. *Energy Environ. Sci.* **7**, 1895–1901 (2014).

13 Zhiping Yan, X. Y., Ali Han, Peng Xu, Pingwu Du. Noble-Metal-Free Ni(OH)_2_-Modified CdS/Reduced Graphene Oxide Nanocomposite with Enhanced Photocatalytic Activity for Hydrogen Production under Visible Light Irradiation. *J. Phys. Chem. C* **118**, 22896–22903 (2014).

14 Zhang, W., Wang, Y., Wang, Z., Zhong, Z. & Xu, R. Highly efficient and noble metal-free NiS/CdS photocatalysts for H2 evolution from lactic acid sacrificial solution under visible light. *Chem. Commun.* **46**, 7631–7633 (2010).
